# Protein complexes detection based on node local properties and gene expression in PPI weighted networks

**DOI:** 10.1186/s12859-021-04543-4

**Published:** 2022-01-06

**Authors:** Yang Yu, Dezhou Kong

**Affiliations:** Software College, Shenyang Normal University, Shenyang, 110034 People’s Republic of China

**Keywords:** Protein complex, Protein–protein interaction (PPI), Resource allocation, Weighted graph construction

## Abstract

**Background:**

Identifying protein complexes from protein–protein interaction (PPI) networks is a crucial task, and many related algorithms have been developed. Most algorithms usually employ direct neighbors of nodes and ignore resource allocation and second-order neighbors. The effective use of such information is crucial to protein complex detection.

**Result:**

Based on this observation, we propose a new way by combining node resource allocation and gene expression information to weight protein network (NRAGE-WPN), in which protein complexes are detected based on core-attachment and second-order neighbors.

**Conclusions:**

Through comparison with eleven methods in Yeast and Human PPI network, the experimental results demonstrate that this algorithm not only performs better than other methods on 75% in terms of f-measure+, but also can achieve an ideal overall performance in terms of a composite score consisting of five performance measures. This identification method is simple and can accurately identify more complexes.

**Supplementary Information:**

The online version contains supplementary material available at 10.1186/s12859-021-04543-4.

## Background

Proteins are the basis of biological activities, and their functions are generally expressed by the interactions between proteins [1]. In organisms, protein–protein interaction (PPI) networks consist of proteins and protein interactions. PPI networks provide an elegant means for expressing gene regulation and metabolic pathways in complex biological systems [2]. Protein complexes are the locally dense regions of PPI networks and possess graph-like structures in which a node represents a protein and an edge represents interaction between two proteins [3].

Complexes take part in many diverse biochemical activities that are fundamental to all kinds of functions, such as cell homeostasis, cell cycle control, growth, and proliferation. Moreover, specific functional modules usually are related to certain diseases.

Although great progress has been made in identifying protein complexes, laboratory-based methods are expensive, ineffective and sometimes even infeasible, and only parts of protein complexes are located. In addition, experiments in the laboratory are often incomplete because of the constraints of experimental conditions. As it is necessary to overcome the lacking of laboratory-based methods, a large number of computational algorithms have been designed as alternative methods to identify protein clusters, such as density-based clustering [4–8], hierarchical clustering[8–10], partition-based clustering [11, 12], flow simulation-based clustering[13–16] and other methods with integrating biological and topological multiple information [17–20]. Although methods of protein complexes detection have achieved some effective results, how to reasonably integrate PPI node local data and gene expression biological information to construct weighted graphs, and how to define effective detection methods to identify complexes from the weighted network still need further study. Only direct neighbors are applied to PPI network clustering problems, which is not sufficient. In fact, node resource allocation information and second-order neighbors often contain some important potential information in PPI networks.

Aiming at the solution for the above-mentioned problems, we introduce a novel method based on resource allocation and gene expression in weighted PPI networks (called NRAGE-WPN) with based on core-attachment structure and second-order neighbors searching. First, based on the resource allocation and gene expression of the PPI network, a new weight metric is designed to accurately describe the interaction between proteins. Then our method detects a series of dense complex cores based on density and network diameter constraints and the final complexes are recognized by expanding the second-order neighbors of nodes in core complexes. This identification method is simple and can accurately identify more complexes.

## Methods

**Fig. 1:**
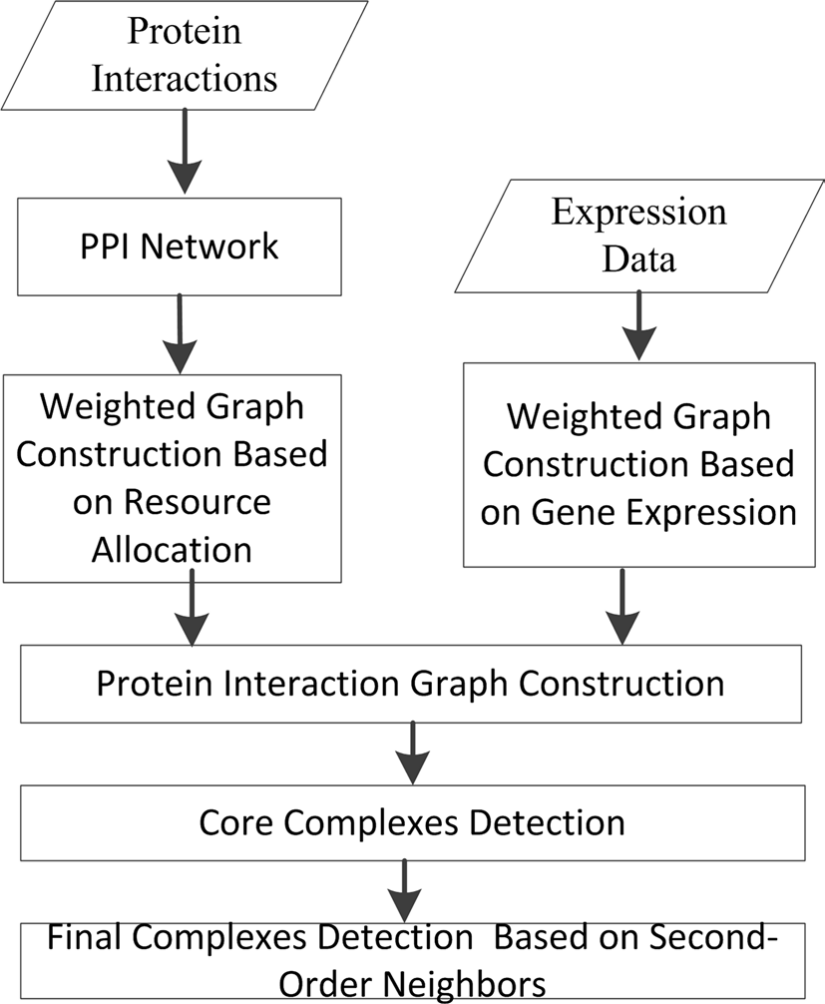
The workflow of our method

Protein complex detection with a computational approach from PPI data is useful as the useful supplement to the limited experimental methods. Besides the enhancement in graph clustering techniques, successful and accurate methods for protein complex prediction depends more on the construction of weighted graphs. Therefore, constructing weighted graph for protein interactions is essential. In this section, we introduce a novel method based on resource allocation and gene expression in weighted PPI networks with two main steps. First, a method is proposed to evaluate the reliability of the protein interaction data considering both the common neighbor information and gene expression profiles through the weighted graph construction. Second, protein complexes are detected based on core-attachment and second-order neighbors in this new weighted graph. The workflow of our method is shown in Fig. 1.

### Assessing the reliability of protein interaction

To represent a PPI network, a 3-element tuple $G = ({\text{V}},E,{\text{W}})$ is employed, where $V = (V_{i} )(1 \le i \le N)$ is a set of N proteins, and $E = \{ e_{ij} \}$ is the set of PPI edges whose values are stored in matrix W. For each pair of nodes, $i,j \in V$ and the edge $e_{ij}$ is assigned a score as $w_{ij}$. Inspired by the reference [21], resource allocation index (RA), is introduced to measure the similarity of interaction proteins in a network and a weighted graph based on resource allocation (WRA) is constructed in this step.

**Fig. 2:**
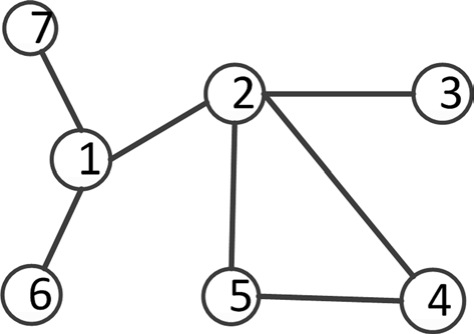
Sample network

Taking Fig. 2 as an example, there is an edge between node 1 and node 2 and no common neighbors between them, but $e_{12}$ is an important bridge for information transmission between node group{1, 2, 6, 7} and node group{1, 2, 3, 4, 5}. Simply, it is assumed that the transmitter 1 can carry resources, and will equally deliver it among all its neighbors. Based on this, the similarity of two nodes is shown in Eq. (1). We can consider node i and node j, which are directly connected without common neighbors and the node i can transmit the information to node j through edge $e_{ij}$ to help the communication between two clusters {1, 2, 6, 7} and {1, 2, 3, 4, 5}. The value range of WRA belongs to [0 1]. This measure requires only the information of the nearest neighbors which therefore has very low computational complexity. $N(i)$ is the set of the neighbors of node i and node i, N(j) is the set of the neighbors of node j and node j.

1 $$WRA_{{{\text{ij}}}} = \sum\limits_{u \in N(i) \cap N(j)} {\frac{1}{{{\text{N}}(u)}}}$$

2 $$W_{N} = \left\{ {WRA_{{{\text{ij}}}} } \right\}$$

### Pearson’s correlation of expression levels

Co-expression genes tend to encode interacting proteins [22]. In this paper, we mainly concentrate on linear gene expression networks unless explicitly stated otherwise and Pearson’s correlation coefficient of expression levels (PCC) is employed as biological information for interacting protein pair p and q. According to GBA principle (i.e. genes with similar expression spectrums have similar biological functions) [23], a higher correlation suggests a higher confidence in their interaction. PCC is generally used to measure the strength of the linear relationship between two variables and is also commonly used to measure the linear relationship between two sets of gene expression values. Suppose there are two columns of gene expression profiles ${\text{X}} = (x_{1} , \ldots ,x_{n} )$ and ${\text{Y}} = ({\text{y}}_{1} , \ldots ,{\text{y}}_{n} )$. Matrix *W*_p_ is formed by the PCC calculation formula, which is defined in Eq. (3). The value range of PCC belongs to [− 1 1]. If PCC (X,Y) < 0, it means that gene X and Y show a negative correlation; if PCC (X,Y) > 0, it means gene X and Y show a positive correlation, PCC (X, Y) = 0 means that there is no correlation between genes X and Y. If PCC(X, Y) < 0, protein pairs will be removed from PPI network in order to reduce the negative effect of low noise data on the detection results of mining protein complexes. The value range [0 1] of PCC is employed in this step.

3 $${\text{PCC}}_{{{\text{ij}}}} = \frac{{\sum\nolimits_{i = 1}^{n} {(x_{i} - \overline{x})(y_{i} - \overline{y})} }}{{\sqrt {\sum\nolimits_{i = 1}^{n} {(x_{i} - \overline{x})^{2} } } \sqrt {\sum\nolimits_{i = 1}^{n} {(y_{i} - \overline{y})^{2} } } }}$$

4 $$W_{{\text{p}}} = \left\{ {PCC_{ij} } \right\}$$

where $\overline{x}$ denotes the average value of the expression value of gene X at 36 different times and $\overline{y}$ denotes the average value of the expression value of gene Y at 36 different times.

### Weighted graph construction

In this part, we first describe how to compute the weighted value by combining gene expression information (GEI) based on PCC and RA information between two interaction proteins. The final weighted construction formula is proposed in Eq. (5).

5 $$W = \alpha W_{{\text{P}}} + (1 - \alpha )W_{{\text{N}}}$$

Matrix $W_{P}$ is constructed based on Pearson correlation coefficient and matrix $W_{N}$ is constructed based on RA, respectively. After a simple calculation, the range of values can be known from 0 to 2. The final values are normalized to [0 1]. $\alpha$($0 \le \alpha \le 1$) is a constant, where a smaller $\alpha$ indicates that the importance of the modules is dependent more on RA information of the network, and a bigger $\alpha$ indicates that the importance of the modules depends more on gene expression information. When $\alpha = 0$, the weighted method only considers RA information. When $\alpha = 1$, the weighted method only considers gene expression information. Therefore the Eq. (5) can measure the differential importance of interaction in protein networks by integrating node local information and biological information.

### Detecting protein complexes in weighted graphs

The proposed algorithm, NRAGE-WPN, consists of two phases: weighted graph construction and core-attachment protein complex detection based on second-order neighbors searching. In the weighted graph construction phase, gene expression information and common neighbor information are integrated. A detailed description of the algorithm is outlined in Algorithm 1. Line 1 is for constructing matrix *W*_*N*_ with the given PPI datasets. Line 2 is for constructing matrix_._ *W*_p_ with the gene expression data. Line 3 is for constructing the new matrix *W* based on *W*_*N*_ and *W*_p_, and the protein interaction confidence is the sum of the weights of *W*_*N*_ and *W*_p_. Lines 4–8 are for identifying core clusters. Lines 9–11 are for enlarging core clusters based on second-order neighbors of nodes in each core.
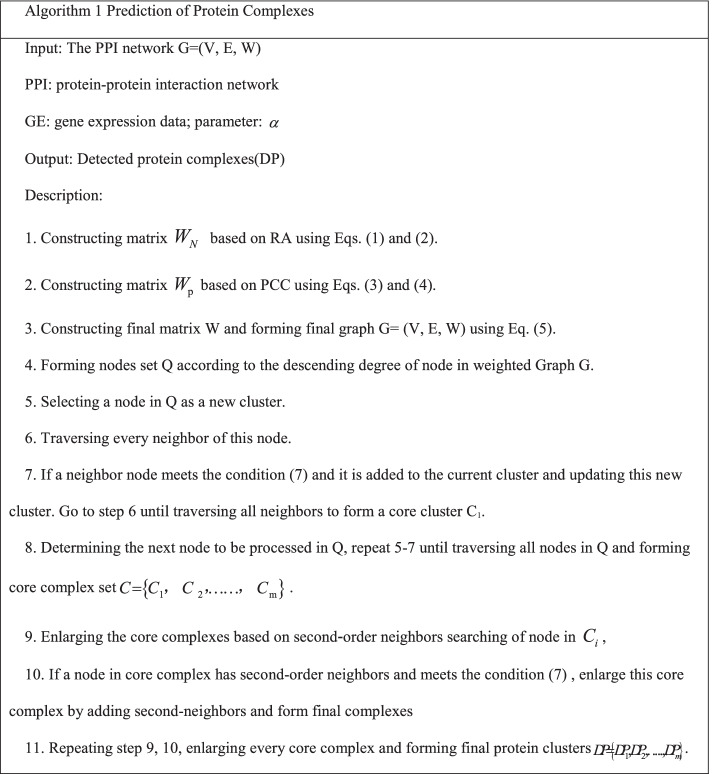


In this algorithm, density and diameter are employed as the condition for complex detection.

If a node meets the two constraints in condition (7), it is added to the current cluster (subgraph). Generally, $\lambda$ is usually set to 0.7 and $\delta$ is set to 2, according to the references [12, 24].

(1) Density: The degree of a node V is the sum of the weights for each edge connecting to this node. Density in the weighted subgraph G = (V, E) is defined in (6). $\left| {\text{N}} \right|$ is the number of nodes in G and w(e) is the weight of the edge $e_{ij}$ in G.

6 $$\begin{aligned} & {\text{m}} = \sum\limits_{{e_{ij} \in E}} {w(e)} \\ & density(G) = \frac{2*m}{{(\left| {\text{N}} \right|*(\left| N \right| - 1))}} \\ \end{aligned}$$

(2) Network Diameter: Diameter is the shortest path in a cluster.

7 $$diameter \le \delta \,and\,density \ge \lambda$$

## Results

### Datasets

The effectiveness of our method is evaluated using PPI networks and gold standards of protein complexes from yeast and human and the detail information is shown in Table 1 and relative detail information can be find in reference [25]. GSE3431 dataset [26] is employed in our paper which records the data of 36 time points during three successive metabolic cycles.

**Table 1:** PPI networks and gold standards in our experiments

	Yeast	Human
PPI networks	Collins [36],Gavin [37], Krogan core [38], Krogan extended [29]	STRING [39], PIPS [40]
Gold standards	CYC2008 [41], MIPS [42]	Corum [43]

### Evaluation criteria

To evaluate our method on benchmark datasets and compare NRAGE-WPN with other methods, evaluation measures are given in this section, such as sensitivity (SN), positive predictive value (PPV), accuracy (ACC), separation (SEP), fraction match (FRM), maximum matching ratio(MMR), precision (Prec), recall (Rec) and f-measure, precision+, recall+, f-measure+, the sum (F_MMR) of MMR and f-measure+, the composite score(CS) of MMR, FRM, SEP, ACC and f-measure [25]. Given a set of benchmark protein complexes $R = \{ R_{1} ,R_{2} , \ldots ,R_{n} \}$ and a set of predicted clusters $P = \{ P_{1} ,P_{2} , \ldots ,P_{n} \}$, two protein complexes, namely, $R_{i}$ and $P_{j}$, are generated from benchmark complex datasets R and predicted protein complex sets P, respectively. $T_{ij}$ is the number of proteins in common between *i*th benchmark complex $R_{i}$ and *j*th predicted complex $P_{j}$. $S{\text{N}}$, PPV and ACC are defined as follows. $N_{i}$ presents the size of proteins in the *i*th benchmark module. Here, n is the number of benchmark complexes and m is the number of predicted complexes.

8 $$S{\text{N}} = \frac{{\sum\nolimits_{i = 1}^{n} {\mathop {\max }\limits_{j} \{ T_{ij} \} } }}{{\sum\nolimits_{i = 1}^{n} {N_{i} } }}\quad PPV = \frac{{\sum\nolimits_{j = 1}^{m} {\mathop {\max \{ T_{ij} \} }\limits_{i} } }}{{\sum\nolimits_{j = 1}^{m} {\sum\nolimits_{i = 1}^{n} {T_{ij} } } }}\quad ACC = \sqrt {Sn \times PPV}$$

To evaluate protein complex prediction in terms of precision and recall, the Jaccard index is employed. The located complex $P_{{\text{j}}}$ is defined to match the real complex $R_{{\text{i}}}$ if the Jacquard similarity is greater than 0.5.

9 $$\begin{array}{*{20}c} {{\text{Jaccard}}(P_{j} ,R_{i} ) = \frac{{\left| {P_{j} \cap R_{i} } \right|}}{{\left| {P_{j} \cup R_{i} } \right|}}} & {precision = \frac{{\left| {\{ P_{j} \in P\left| {\exists R_{i} \in R,P_{j} matches} \right.R_{i} \} } \right|}}{m}} \\ {recall = \frac{{\left| {\{ R_{i} \in R\left| {\exists P_{j} \in P,P_{j} matchesR_{i} } \right.\} } \right|}}{n}} & {f - measure = \frac{2*recall*precision}{{recall + precision}}} \\ \end{array}$$

In terms of precision+, recall+ and f-measure+, neighborhood affinity score $NA(P_{j} ,R_{i} )$ between $P_{j}$ and $R_{i}$, as defined in Eq. (10) can be used to determine whether they match with each other. If $NA(P_{j} ,R_{i} )$ = ω, $\omega \ge t$, $\omega$ is greater than 0.2, $P_{j}$ and $R_{i}$ are considered to be matching. In this paper, t is usually set as 0.20. $\left|{\mathrm{P}}_{\mathrm{i}}\right|$ $\left| {P_{i} } \right|$ and $\left|{\mathrm{R}}_{\mathrm{j}}\right|$ $\left| {R_{j} } \right|$ are the numbers of proteins in ${\mathrm{P}}_{\mathrm{i}}$ $P_{i}$ and $R_{j}$, respectively.

10 $$NA(P_{j} ,R_{i} ) = \frac{{\left| {P_{j} \cap R_{i} } \right|^{2} }}{{\left| {P_{j} } \right| * \left| {R_{i} } \right|}}$$

11 $$recall^{ + } = \frac{{|\{ R_{j} |R_{j} \in R \wedge P_{i} \in P,P_{i} matchesR_{j} \} |}}{n}\quad precision^{ + } = \frac{{|\{ P_{i} |P_{i} \wedge R_{j} \in R,R_{j} matchesP_{i} \} |}}{m}\quad f - measure^{ + } = \frac{{2*recall^{ + } *precision^{ + } }}{{recall^{ + } + precision^{ + } }}$$

### Comparison with other methods

**Fig. 3:**
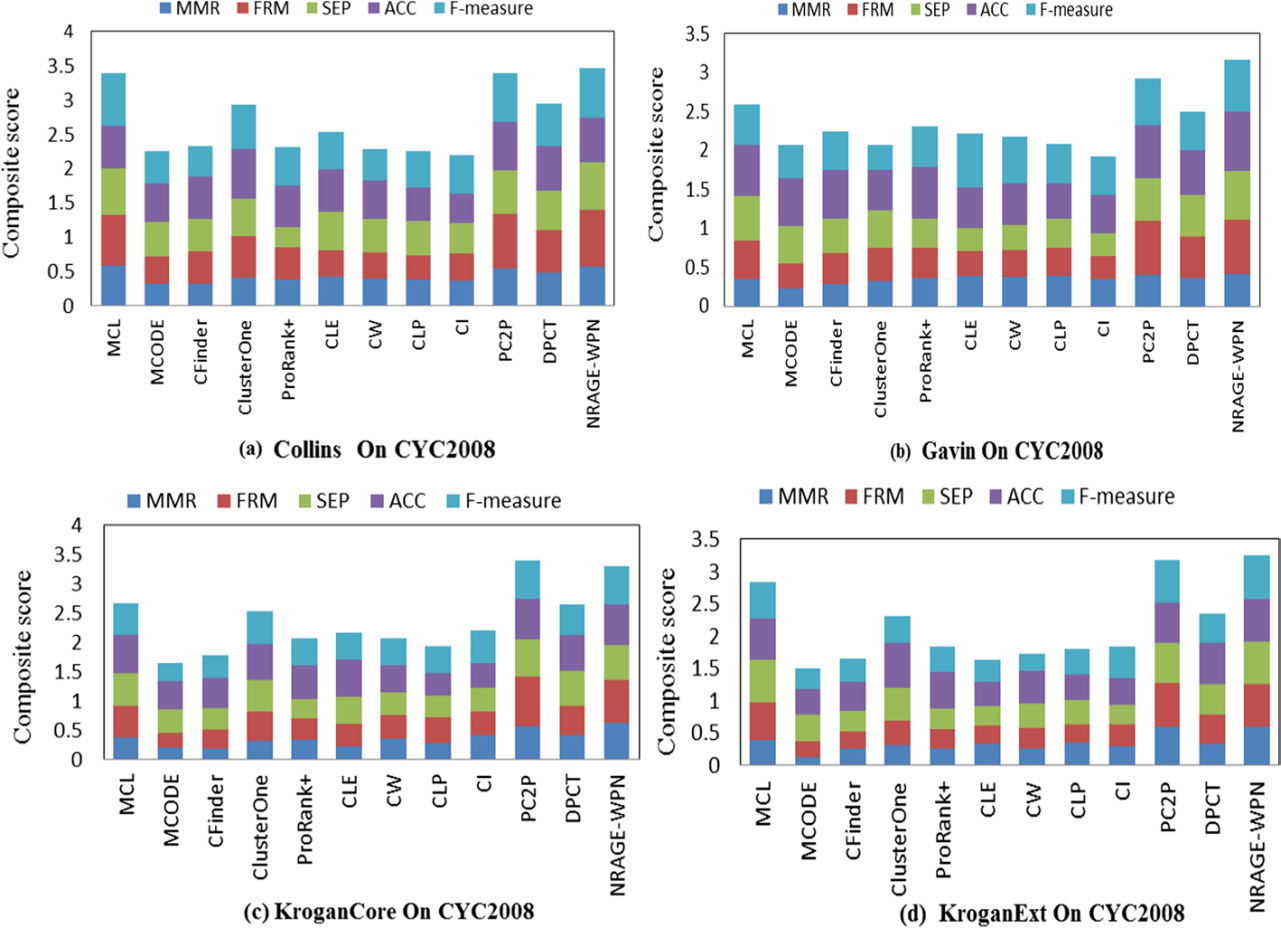
Comparisons of four yeast PPI networks on CYC2008

To inspect the performance of our proposed algorithm, we compare our algorithm with MCODE [6], Cfinder [4], ClusterOne [20], ProRank+ [27], MCL [28], PC2P [25], CLE [7], CW [8], CLP [29], CI [13], DPCT [30] in different measures as shown in Additional file 1: Table S1 and all the weighted graphs are constructed based on Eq. (5). Comparison results about CS measure in four PPI networks of Yeast on CYC2008 are shown in Fig. 3.

Comparative analysis is performed with the sum score of MMR, FMR, SEP, ACC and f-measure. Performances among different methods are compared for yeast and human with the corresponding complex datasets and PPI networks. First, as is illustrated in Fig. 3 that NRAGE-WPN can achieve best performance in Collins, Gavin and KroganExt network and perform better than other ten methods except PC2P in KroganCore in terms of CS on CYC2008. On MIPS, NRAGE-WPN outperforms all methods on MIPS in network Collins and ten methods in network Gavin, KroganExt and KroganCore except PC2P in Additional file 1: Table S1. On CORUM in 2 combinations, NRAGE-WPN can achieve best performances in terms of CS. Second, in terms of f-measure+, NRAGE-WPN results the best performance except in Collins on MIPS. Third, in the rest measures, NRAGE-WPN performs better than most other methods and the all detail information can be shown in Additional file 1: Table S1.

### Assessment performances of f-measure+ and accuracy with parameter α

**Fig. 4:**
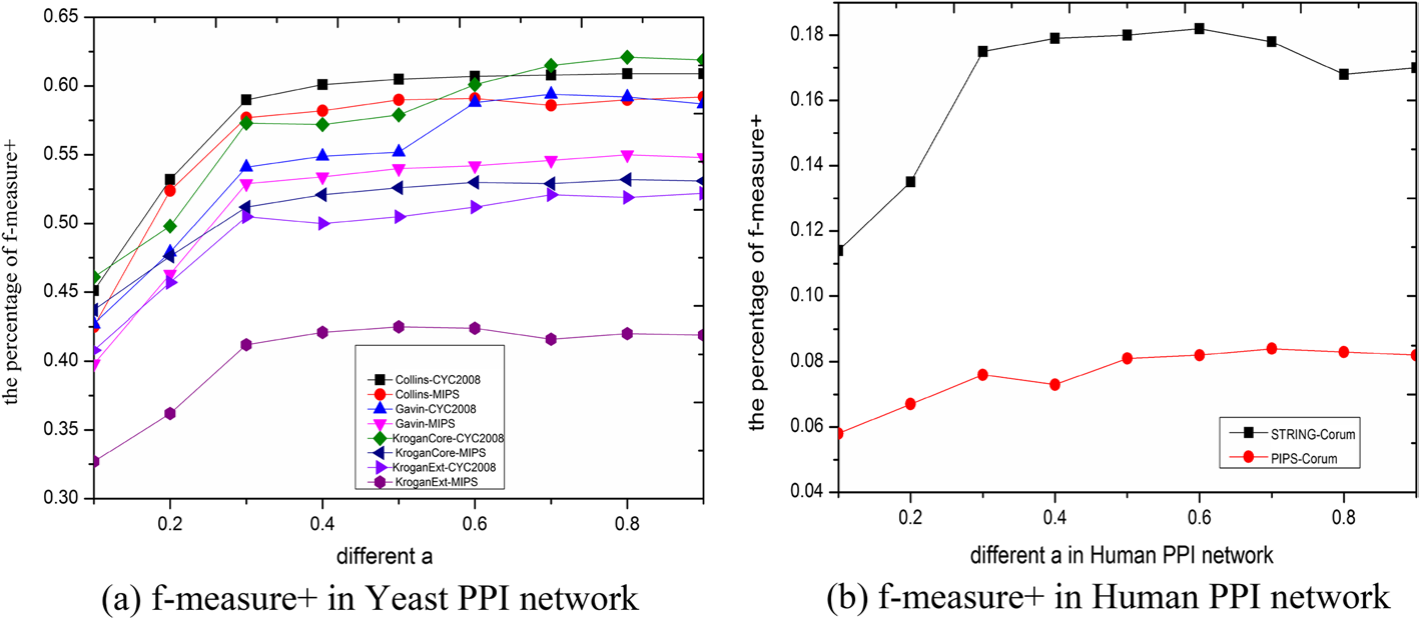
f-measure+ in yeast and human for different parameter *α*

**Fig. 5:**
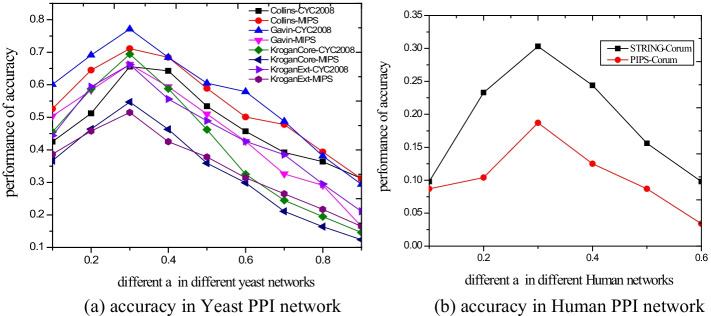
Accuracy yeast and human for different parameter *α*

By evaluating the importance of parameter $\alpha$, we can more intuitively observe the influence of a certain parameter on the experimental results, and it is helpful to understand the advantages and disadvantages of the algorithm and enhance it. The critical parameter $\alpha$ in our method is mainly employed to show the effectiveness of information fusion from local neighbors and gene expression information and to affect the detection results of protein complexes. This experiment investigates the effects of different parameters $\alpha$ from 0.1 to 0.9 at interval of 0.1 on complex detection performance. Using f-measure+ and accuracy as our experimental evaluation criterion, the performances with different $\alpha$ are evaluated as shown in the Figs. 4 and 5, respectively. In Fig. 4,when the parameter $\alpha$ is greater than or equal to 0.3, the f-measure+ tends to stable. In In Fig. 5, when $\alpha$ = 0.3, the best performance of accuracy can be achieved. In this article, we take $\alpha$ = 0.3.

### Robustness to the different thresholds (t)

**Fig. 6:**
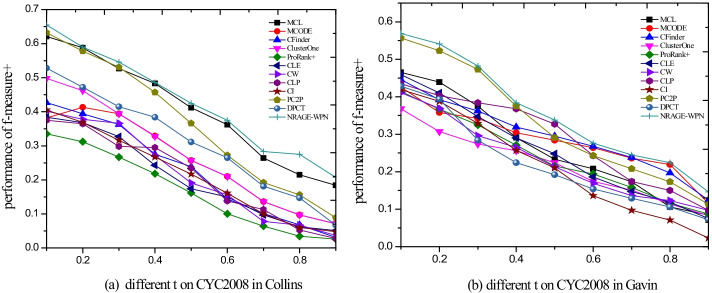
f-measure+ in yeast for different threshold t

In order to illustrate the comprehensive performance of NRAGE-WPN, we demonstrate f-measure+ performances with nine thresholds $t = \left\{ {0.1,0.2,0.3,0.4,0.5,0.6,0.7,0.8,0.9} \right\}$ among different methods in Fig. 6. Figure 6a shows the comparisons of f-measure+ performances on the CYC2008 benchmark dataset in Collins. It can be illustrated that NRAGE-WPN outperforms other eleven methods. Similar results can also be found on the CYC2008 benchmark in Gavin in Fig. 6b. Other comparisons are shown in Additional file 1: Fig. S1, which illustrates that NRAGE-WPN performs better than other combinations on 50%. This further demonstrates the effectiveness of the fusion information from local node and gene expression data.

## Discussion

### Functional analysis

For the protein complexes identified by the NRAGE-WPN algorithm, we measure the effectiveness of the algorithm quantitatively and qualitatively. We analyze the biological significance of the identified protein complexes. Real protein complexes often present high functional homogeneity, so the function enrichment test is employed to demonstrate the biological significances of detected protein complexes [31]. The function enrichment analysis of protein complexes identified from yeast PPI network is carried out to further verify the effectiveness of NRAGE-WPN algorithm. The analysis and comparison of *P* value are shown in Table 2. *P* value of each complex can be divided into one of four intervals from small to large: < E-15, [E-15, E-10], [E-10, E-5], [E-5, 0.001]. When *P* value is greater than 0.001, it is generally considered that the function of the complex is very likely to be randomly assigned and has no biological significance. The percentages in brackets in Table 2 indicate the ratio of the number of complexes in a certain interval to the number of complexes in all intervals. For example, a total of 325 complexes are predicted by NRAGE-WPN on CYC2008 in Collins and effective percentage of NRAGE-WPN is greater than other eleven algorithms. Further, with respect to the biological relevance, the enrichment score of the annotations are employed to evaluate the performance of predicted complex. The average of detected complexes with at least one enriched annotation over all clusters among eleven approaches on six datasets is compared in Additional file 1: Table S2. The results illustrate that NRAGE-WPN predicts biologically relevant clusters with enrichment scores with the top 70% of other methods in terms of the different GO categories.

**Table 2:** Performance of functional enrichment comparison and their *P* values in Collins on CYC2008

Methods	Clusters	Effective (%)	< E-15 (%)	E-15-E-5 (%)	E-5-0.001 (%)
MCL	212	86.46	13.25	34.00	39.21
MCODE	84	89.65	15.50	59	14.75
CFinder	73	83.50	24.25	42.45	16.80
ClusterOne	106	91.85	29.75	52.47	9.63
ProRank+	385	87.52	18.17	48.12	21.23
CLE	215	84.14	35.36	26.43	22.35
CW	164	92.79	19.24	54.31	19.24
CLP	207	94.44	8.79	60.28	25.37
CI	132	92.06	25.35	57.47	9.24
PC2P	283	93.20	43.80	32.65	16.75
DPCT	274	94.05	40.72	37.95	15.38
NRAGE-WPN	325	96.42	41.25	48.75	6.42

### Effectiveness of RA

**Fig. 7:**
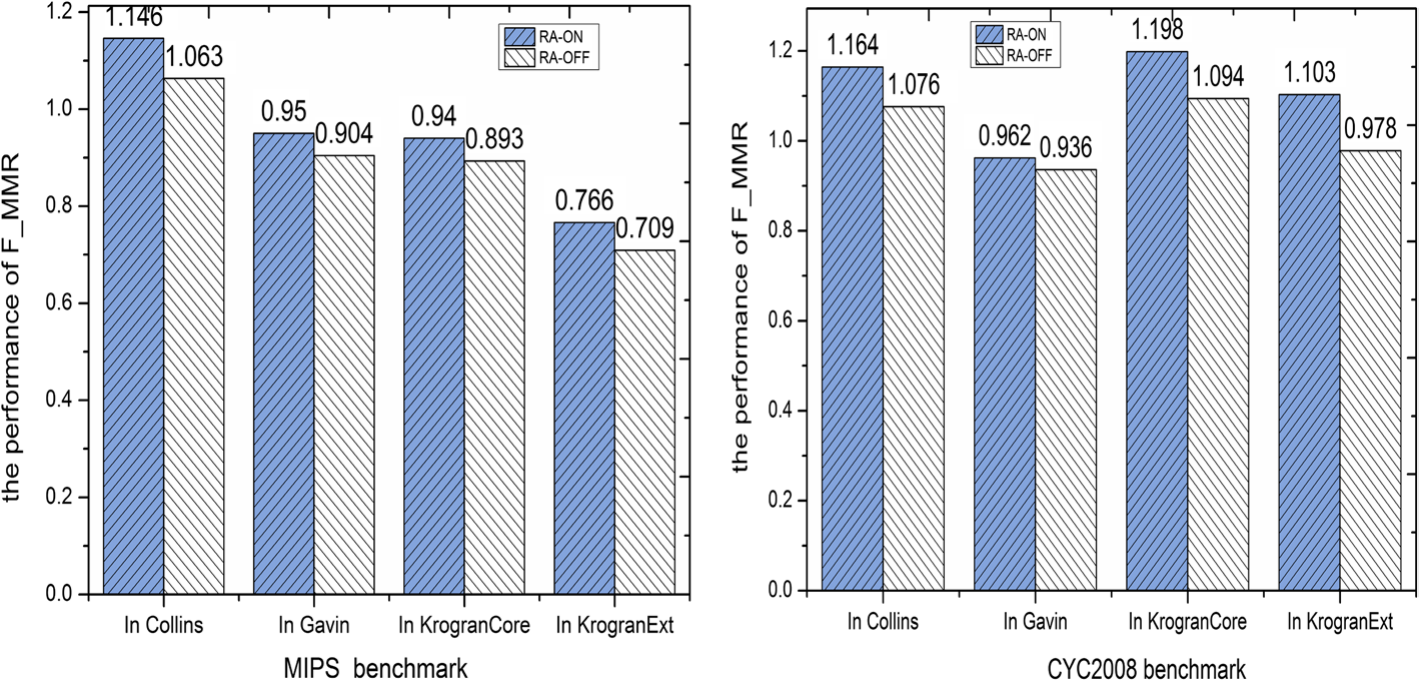
The effectiveness of NRAGE-WPN when RA is off/on with CYC2008 and MIPS benchmarks

Due to the noise data in the PPI network, NRAGE-WPN uses gene expression and RA information to score a weight to each interaction of the PPI network. To assess the effect of using RA in the f-measure+ for complexes detection, we conduct NRAGE-WPN without considering RA information and compare its results with normal the NRAGE-WPN which employs both gene expression and RA information. Without using RA situation, a weighted PPI network is constructed by gene expression only. Figure 7 shows the results of NRAGE-WPN in RA-OFF and RA-ON in Collins, Gavin, KroganCore and KroganExt datasets with CYC2008 and MIPS benchmarks, respectively. From Fig. 7, it can be shown that by introducing RA, the quality performance of F_MMR is enhanced. In term of RA-ON mode in Collins data, F_MMR increases 8.8% for the CYC2008 benchmark and 8.3% for the MIPS benchmark. According to Fig. 7, the same trend can also be shown in other three PPI datasets on two benchmarks, respectively. This experiment shows that using RA can reduce noise data and improve the overall performance of complexes detection.

### Effectiveness of second-order neighbors searching (SNS)

**Fig. 8:**
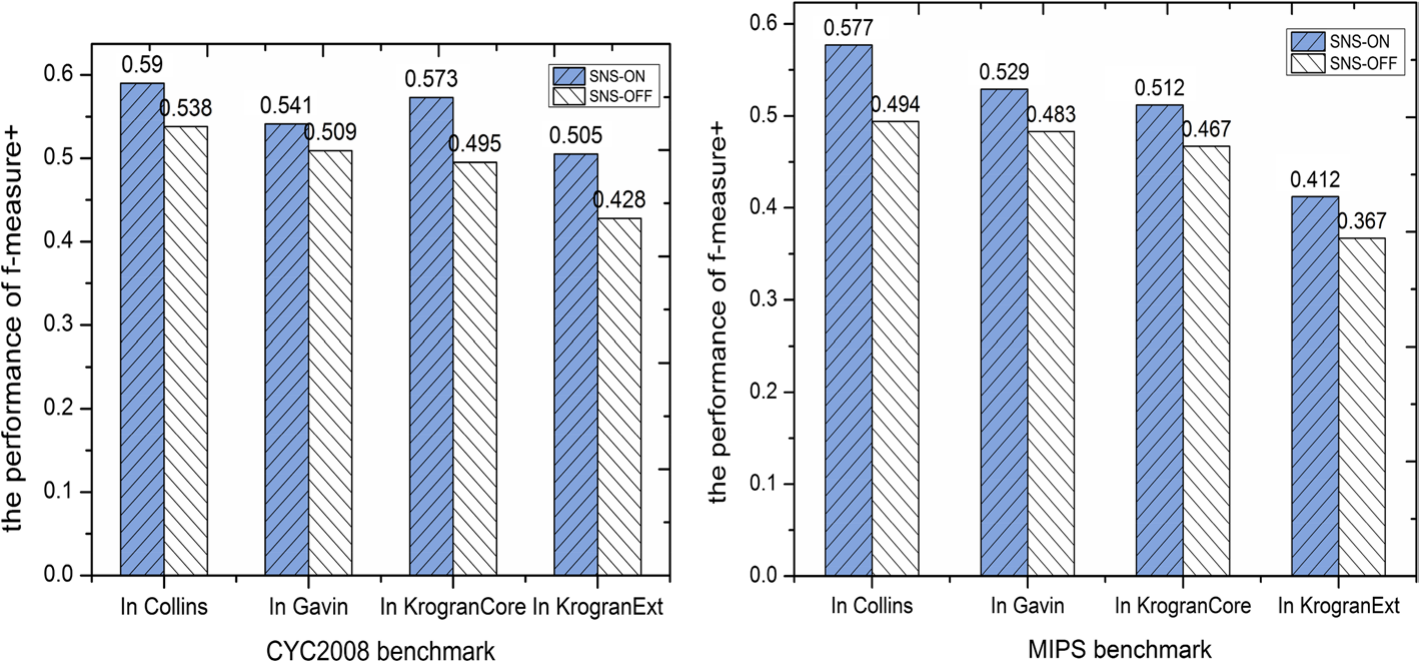
The effectiveness of using SNS in NRAGE-WPN compare with NRAGE-WPN without using SNS in four PPI datasets with CYC2008 and MIPS benchmarks

The second phase of the NRAGE-WPN method is to enlarge the core complexes by second-order neighbors. After detecting core protein complexes from weighted PPI network, due to the nature of complexes of core-attachment, there may be many attachment parts to be added to the cores. In this situation, the cores and attachment parts are combined to form final complexes. In order to assess the effect of introducing second-order neighbors searching(SNS), we conduct NRAGE-WPN without its second phase. Figure 8 shows the comparison between second-order neighbors searching-on (SNS-ON) and second-order neighbors searching-off (SNS-OFF) modes in terms of f-measure+. On the CYC2008 benchmark, when NRAGE-WPN uses the SNS phase, we can see a 5.2%, 3.2%, 7.8% and 7.7% rise in Collins, Gavin, KroganCore, KroganExt, respectively. As the results show, performance of f-measure+ can be improved by introducing the second-order neighbors searching.

### Assessment of density in different weighted graphs

12 $$W = \alpha W_{{\text{P}}} + (1 - \alpha ){\text{KBRV}}$$

**Fig. 9:**
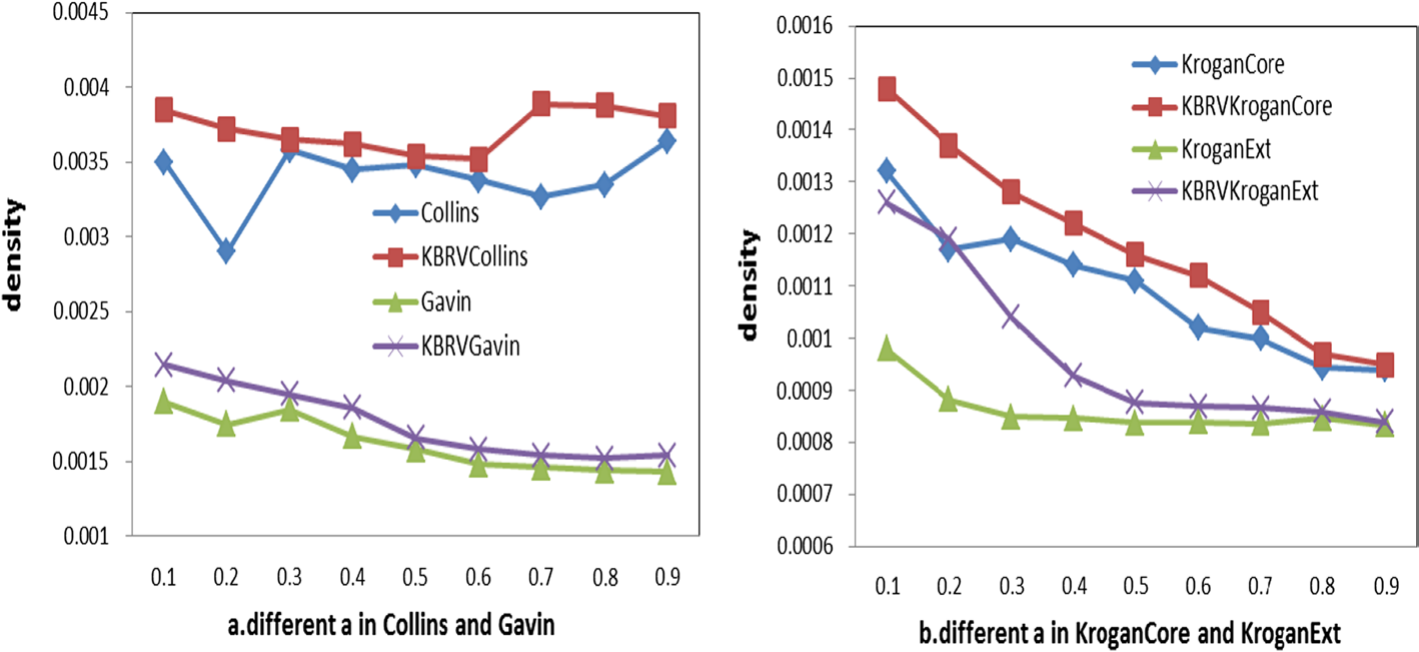
The density of using different a by comparing with KVRB method for construction of four weighted graph in Yeast

Although PCC cannot identify whether gene variables are directly regulated or indirectly regulated [33–35], in this paper, we mainly focus on PCC as biological information to construct weighted graph network based on gene expression, which is one of the most commonly used methods for constructing gene regulatory networks. At the same time, we discuss the influence of nonlinear correlation of gene expression on the density of whole network. We construct another four weighted graphs based on KBRV [32] method and the density of networks are compared in Fig. 9. First, the results show that four weighted networks based on KBRV can increase the density of PPI network. The reason is that the weighted value of the protein pairs that can be increased by (12). Second, we can find that when $\alpha$ belongs to [0.3 0.5], the densities of four weighted graph by (5) decrease slow. In our experiment, $\alpha$ = 0.3 is used. Lastly, in our future work,we will focus on the nonlinear correlation of gene expression for weighted graph construction and complex detection.

## Conclusions

The identification of protein complexes is important for discovering and understanding the cellular organizations and biological processes in PPI networks. In this paper a new approach named NRAGE-WPN is proposed for identifying protein complexes in protein–protein interaction networks. Based on the resource allocation and gene expression of the PPI network, we first design a new weight metric to accurately describe the interaction between proteins. Our method then constructs a series of dense complex cores based on density and network diameter constraints, and the final complexes are recognized by expanding the second-order neighbors of nodes in core complexes. Through comparison with eleven methods in Yeast and Human PPI network, the experimental results demonstrate that this algorithm not only performs better than other methods on 75% in terms of f-measure+, but also can achieve an ideal overall performance in terms of a composite score consisting of five performance measures. In the future work, we will focus on locating sparse and density protein complexes by integrating multiple information.

## Supplementary Information

**Additional fle 1.** The Additional fle 1 contains Figures 1, Tables 1 and 2. **Figure 1** shows comparative analysis of approaches for prediction of protein complexes in Yeast on different threshold t. **Table 1** shows Comparative analysis of eleven algorithms with respect to different measures. **Table 2** shows the average of enrichment score of predicted complexes with at least one enrichedannotation over all clusters compared among eleven methods across six datasets.

## Data Availability

The datasets are available at https://github.com/graceyy000/dataset and NRAGE-WPN codes are available from the corresponding author on reasonable request.

## Abbreviations

PPI: Protein–protein interaction
NRAGE-WPN: A new way by combining node resource allocation and gene expression information to weight protein network
RA: Resource allocation
WRA: Weighted graph based on resource allocation
PCC: Pearson’s correlation coefficient
GEI: Gene expression information
DP: Detected protein complexes
SN: Sensitivity
PPV: Positive predictive value
ACC: Accuracy
SEP: Separation
FRM: Fraction match
MMR: Maximum matching ratio
Prec: Precision
Rec: Recall
F_MMR: The sum of MMR and f-measure+
CS: The composite score of MMR, FRM, SEP, ACC and f-measure
