# Supplementary material for: Protein complexes detection based on node local properties and gene expression in PPI weighted networks
Source: BMC Bioinformatics. 2022 Jan 6;23:24. doi: 10.1186/s12859-021-04543-4 (PMC8734347; doi:10.1186/s12859-021-04543-4)
Supplement: Supplementary file 1 — Additional fle 1. The Additional fle 1 contains Figures 1, Tables 1 and 2. Figure 1 shows comparative analysis of approaches for prediction of protein complexes in Yeast on different threshold t. Table 1 shows Comparative analysis of eleven algorithms with respect to different measures. Table 2 shows the average of enrichment score of predicted complexes with at least one enrichedannotation over all clusters compared among eleven methods across six datasets. [file 12859_2021_4543_MOESM1_ESM.pdf]

Supplementary Figure1 Comparative analysis of approaches for prediction of protein complexes in Yeast on different threshold t.

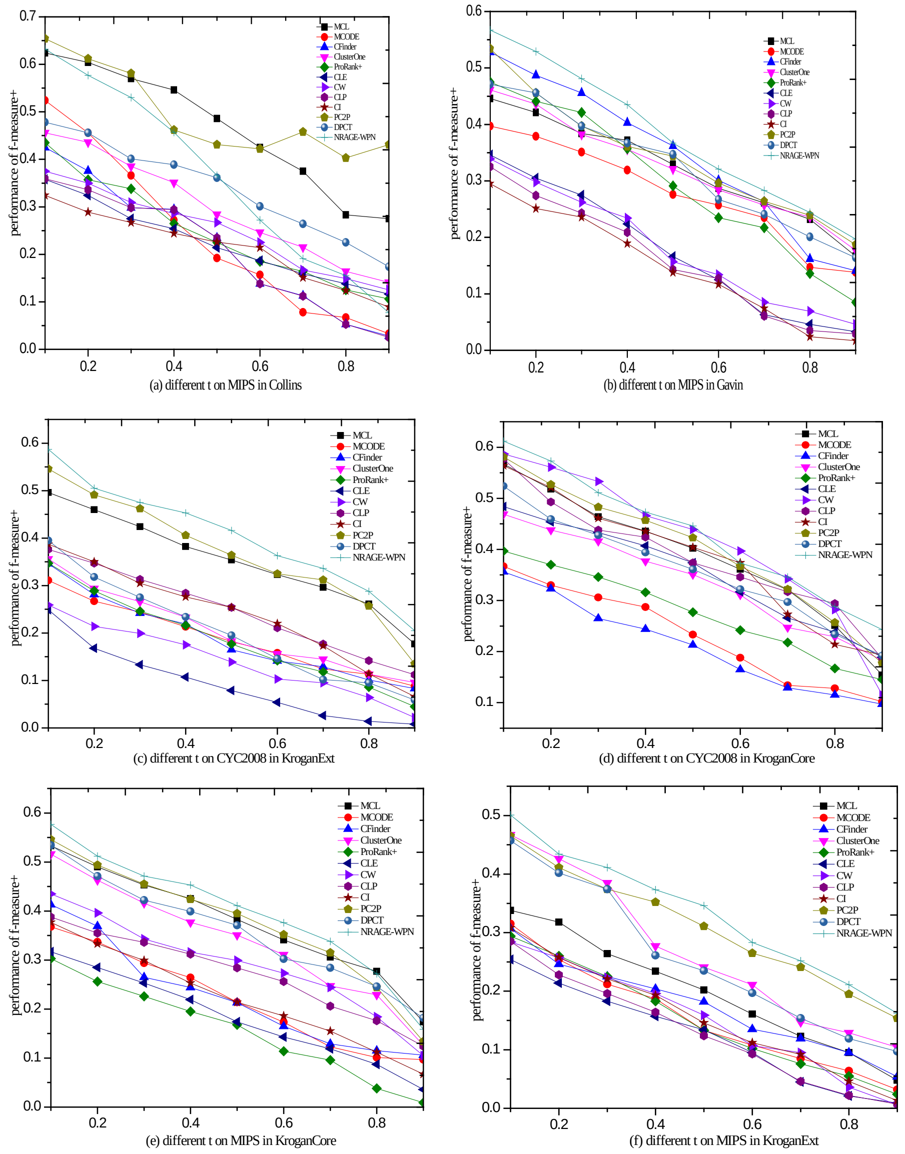

Supplementary Table 1 Comparative analysis of eleven algorithms with respect to different measures

Comparative analysis Yeast-CYC2008																	
dataset	mehtods	#clusters	MMR	FRM	SEP	PPV	SN	ACC	Prec	Rec	F-measure	Prec+	Rec+	F-measure	F_MMR	Modularity	Composite Score
	MCL	212	0.587	0.74	0.679	0.675	0.579	0.625	0.874	0.683	0.767	0.763	0.478	0.588	1.175	0.799	3.398
	MCODE	84	0.325	0.396	0.498	0.543	0.587	0.565	0.864	0.318	0.465	0.819	0.276	0.413	0.738	0.604	2.249
	CFinder	73	0.315	0.482	0.468	0.506	0.747	0.615	0.781	0.321	0.455	0.815	0.26	0.394	0.709	0.751	2.335
Collins	ClusterOn	106	0.406	0.607	0.547	0.626	0.846	0.728	0.874	0.511	0.645	0.795	0.326	0.462	0.868	0.782	2.933
	ProRank+	385	0.375	0.476	0.294	0.468	0.805	0.614	0.694	0.459	0.553	0.261	0.387	0.312	0.687	0.307	2.312
	CLE	215	0.427	0.385	0.564	0.527	0.709	0.611	0.739	0.428	0.542	0.419	0.328	0.368	0.795	0.726	2.529
	CW	164	0.391	0.394	0.482	0.471	0.674	0.563	0.574	0.377	0.455	0.509	0.301	0.378	0.769	0.625	2.285
	CLP	207	0.375	0.364	0.497	0.395	0.587	0.482	0.658	0.461	0.542	0.476	0.296	0.365	0.74	0.713	2.26
	CI	132	0.367	0.401	0.439	0.382	0.496	0.435	0.588	0.528	0.556	0.526	0.284	0.369	0.736	0.625	2.198
	PC2P	283	0.549	0.798	0.634	0.644	0.762	0.7	0.765	0.674	0.717	0.645	0.524	0.578	1.127	**0.76**	3.398
	DPCT	274	0.482	0.618	0.574	0.597	0.728	0.659	0.639	0.594	0.616	0.548	0.613	0.579	1.061	**0.72**	2.949
	NRAGE-W	325	0.574	0.824	0.689	0.587	0.734	0.656	0.727	0.725	0.726	0.687	0.517	0.59	1.164	0.747	3.469
dataset	mehtods	#clusters	MMR	FRM	SEP	PPV	SN	ACC	Prec	Rec	F-measure	Prec+	Rec+	F-measure	F_MMR	Modularity	Composite Score
	MCL	135	0.342	0.496	0.572	0.523	0.897	0.661	0.686	0.425	0.525	0.642	0.334	0.439	0.781	0.823	2.596
	MCODE	96	0.228	0.317	0.481	0.564	0.671	0.613	0.745	0.304	0.432	0.694	0.241	0.358	0.586	0.621	2.071
	CFinder	117	0.278	0.407	0.437	0.523	0.795	0.631	0.813	0.348	0.487	0.725	0.269	0.392	0.67	0.774	2.24
Gavin	ClusterOn	137	0.315	0.429	0.482	0.415	0.725	0.528	0.308	0.325	0.316	0.314	0.301	0.307	0.622	0.801	2.07
	ProRank+	97	0.356	0.39	0.376	0.567	0.825	0.672	0.698	0.405	0.513	0.354	0.382	0.367	0.723	0.764	2.307
	CLE	75	0.382	0.326	0.289	0.483	0.581	0.527	0.763	0.632	0.691	0.386	0.438	0.41	0.792	0.586	2.215
	CW	87	0.375	0.349	0.312	0.467	0.625	0.535	0.624	0.592	0.608	0.325	0.426	0.369	0.744	0.467	2.179
	CLP	164	0.391	0.361	0.375	0.408	0.512	0.454	0.537	0.465	0.498	0.425	0.384	0.403	0.794	0.382	2.079
	CI	119	0.352	0.296	0.289	0.346	0.874	0.496	0.512	0.481	0.496	0.375	0.401	0.388	0.74	0.397	1.929
	PC2P	194	0.398	0.695	0.549	0.684	0.67	0.677	0.645	0.567	0.603	0.583	0.475	0.523	0.921	0.674	2.922
	DPCT	157	0.364	0.531	0.529	0.467	0.764	0.58	0.607	0.406	0.487	0.392	0.425	0.408	0.772	0.824	2.491

	NRAGE-W	287	0.421	0.684	0.625	0.714	0.837	0.771	0.834	0.549	0.662	0.598	0.494	0.541	0.962	0.598	3.163
dataset	mehtods	#clusters	MMR	FRM	SEP	PPV	SN	ACC	Prec	Rec	F-measure	Prec+	Rec+	F-measure	F_MMR	Modularity	Composite Score
	MCL	158	0.382	0.529	0.568	0.546	0.764	0.646	0.732	0.432	0.543	0.698	0.412	0.518	0.9	0.892	2.668
	MCODE	58	0.205	0.237	0.406	0.601	0.406	0.494	0.625	0.201	0.304	0.617	0.225	0.33	0.535	0.687	1.646
	CFinder	81	0.189	0.312	0.364	0.463	0.608	0.531	0.749	0.265	0.391	0.723	0.208	0.323	0.512	0.825	1.787
KroganCo	ClusterOne	159	0.325	0.495	0.532	0.587	0.665	0.625	0.682	0.465	0.553	0.601	0.345	0.438	0.763	0.743	2.53
	ProRank+	289	0.337	0.362	0.335	0.543	0.584	0.563	0.668	0.372	0.478	0.423	0.329	0.37	0.707	0.521	2.075
	CLE	76	0.229	0.385	0.462	0.703	0.564	0.63	0.584	0.365	0.449	0.528	0.398	0.454	0.683	0.601	2.155
	CW	95	0.352	0.406	0.386	0.439	0.497	0.467	0.512	0.426	0.465	0.603	0.524	0.561	0.913	0.635	2.076
	CLP	146	0.287	0.432	0.364	0.386	0.396	0.391	0.413	0.511	0.457	0.568	0.436	0.493	0.78	0.594	1.931
	CI	94	0.421	0.393	0.413	0.395	0.462	0.427	0.487	0.623	0.547	0.592	0.465	0.521	0.942	0.685	2.201
	PC2P	365	0.573	0.846	0.624	0.702	0.696	0.699	0.598	0.712	0.65	0.546	0.51	0.527	1.1	0.801	3.392
	DPCT	184	0.405	0.512	0.584	0.601	0.649	0.625	0.535	0.511	0.523	0.591	0.429	0.497	0.902	0.594	2.649
	NRAGE-W	337	0.625	0.738	0.596	0.684	0.704	0.694	0.612	0.684	0.646	0.584	0.563	0.573	1.198	0.698	3.299
dataset	mehtods	#clusters	MMR	FRM	SEP	PPV	SN	ACC	Prec	Rec	F-measure	Prec+	Rec+	F-measure	F_MMR	Modularity	Composite Score
	MCL	179	0.382	0.601	0.641	0.523	0.813	0.652	0.625	0.509	0.561	0.538	0.401	0.46	0.842	0.625	2.837
	MCODE	69	0.129	0.234	0.425	0.527	0.298	0.396	0.634	0.214	0.32	0.464	0.187	0.267	0.396	0.384	1.504
	CFinder	52	0.265	0.265	0.321	0.351	0.582	0.452	0.657	0.246	0.358	0.297	0.267	0.281	0.546	0.337	1.661
KroganExt	ClusterOne	159	0.312	0.382	0.512	0.637	0.738	0.686	0.725	0.298	0.422	0.371	0.243	0.294	0.606	0.415	2.314
	ProRank+	254	0.265	0.293	0.325	0.601	0.516	0.557	0.598	0.304	0.403	0.308	0.272	0.289	0.554	0.332	1.843
	CLE	68	0.329	0.285	0.312	0.439	0.316	0.372	0.405	0.277	0.329	0.298	0.117	0.168	0.497	0.354	1.627
	CW	84	0.259	0.324	0.365	0.524	0.497	0.51	0.387	0.198	0.262	0.294	0.168	0.214	0.473	0.298	1.72
	CLP	132	0.346	0.289	0.384	0.367	0.429	0.397	0.519	0.304	0.383	0.416	0.297	0.347	0.693	0.425	1.799
	CI	117	0.297	0.346	0.284	0.451	0.385	0.417	0.614	0.406	0.489	0.387	0.319	0.35	0.647	0.358	1.833
	PC2P	376	0.602	0.678	0.613	0.597	0.654	0.625	0.638	0.701	0.668	0.484	0.498	0.491	1.093	0.512	3.186
	DPCT	179	0.339	0.439	0.488	0.659	0.609	0.634	0.789	0.314	0.449	0.411	0.359	0.383	0.722	0.449	2.349
	NRAGE-W	348	0.598	0.667	0.654	0.638	0.686	0.662	0.657	0.689	0.673	0.499	0.512	0.505	1.103	0.497	3.254

Comparative analysis Yeast-MIPS

dataset	mehtods	#clusters	MMR	FRM	SEP	PPV	SN	ACC	Prec	Rec	F-measure	Prec+	Rec+	F-measure	F_MMR	Modularity	Composite Score
	MCL	146	0.508	0.765	0.628	0.584	0.795	0.681	0.825	0.705	0.76	0.793	0.488	0.604	1.112	0.925	3.342
	MCODE	54	0.278	0.512	0.459	0.524	0.601	0.561	0.749	0.258	0.384	0.714	0.335	0.456	0.734	0.745	2.194
	CFinder	71	0.301	0.461	0.448	0.501	0.761	0.617	0.778	0.364	0.496	0.767	0.249	0.376	0.677	0.817	2.323
Collins	ClusterOne	95	0.498	0.487	0.495	0.494	0.801	0.629	0.847	0.398	0.542	0.815	0.298	0.436	0.934	0.794	2.651
	ProRank+	335	0.264	0.516	0.308	0.462	0.697	0.567	0.767	0.412	0.536	0.367	0.347	0.357	0.621	0.579	2.191
	CLE	89	0.314	0.347	0.391	0.381	0.425	0.402	0.385	0.297	0.335	0.467	0.248	0.324	0.638	0.307	1.789
	CW	102	0.295	0.259	0.364	0.394	0.513	0.45	0.367	0.281	0.318	0.513	0.266	0.35	0.645	0.228	1.686
	CLP	146	0.134	0.384	0.401	0.267	0.467	0.353	0.408	0.218	0.284	0.354	0.319	0.336	0.47	0.435	1.556
	CI	107	0.167	0.367	0.364	0.463	0.385	0.422	0.376	0.307	0.338	0.295	0.284	0.289	0.456	0.267	1.658
	PC2P	196	0.546	0.744	0.701	0.624	0.747	0.683	0.835	0.597	0.696	0.668	0.564	0.612	1.158	0.829	3.37
	DPCT	108	0.522	0.549	0.528	0.612	0.749	0.677	0.769	0.439	0.559	0.624	0.337	0.438	0.96	0.825	2.835
	NRAGE-W	176	0.569	0.762	0.724	0.658	0.768	0.711	0.817	0.566	0.669	0.654	0.516	0.577	1.146	0.861	3.435

dataset	mehtods	#clusters	MMR	FRM	SEP	PPV	SN	ACC	Prec	Rec	F-measure	Prec+	Rec+	F-measure	F_MMR	Modularity	Composite Score
	MCL	105	0.317	0.534	0.558	0.461	0.884	0.638	0.717	0.284	0.407	0.682	0.304	0.421	0.738	0.936	2.454
	MCODE	68	0.251	0.419	0.462	0.485	0.659	0.565	0.805	0.364	0.501	0.652	0.267	0.379	0.63	0.697	2.198
	CFinder	71	0.189	0.459	0.538	0.446	0.812	0.602	0.728	0.267	0.391	0.734	0.364	0.487	0.676	0.816	2.179
Gavin	ClusterOne	123	0.311	0.612	0.467	0.368	0.835	0.554	0.598	0.405	0.483	0.691	0.318	0.436	0.747	0.562	2.427
	ProRank+	325	0.361	0.538	0.338	0.528	0.768	0.637	0.628	0.327	0.43	0.456	0.427	0.441	0.802	0.346	2.304
	CLE	72	0.293	0.348	0.297	0.439	0.536	0.485	0.523	0.295	0.377	0.315	0.295	0.305	0.598	0.429	1.8
	CW	87	0.227	0.397	0.367	0.365	0.612	0.473	0.498	0.228	0.313	0.338	0.267	0.298	0.525	0.367	1.777
	CLP	109	0.198	0.267	0.285	0.431	0.394	0.412	0.567	0.195	0.29	0.219	0.367	0.274	0.472	0.357	1.452
	CI	85	0.319	0.468	0.357	0.384	0.468	0.424	0.346	0.308	0.326	0.278	0.197	0.231	0.55	0.318	1.894
	PC2P	148	0.467	0.695	0.628	0.572	0.785	0.67	0.758	0.478	0.586	0.598	0.368	0.456	0.923	0.768	3.046
	DPCT	159	0.349	0.576	0.538	0.402	0.817	0.573	0.527	0.454	0.488	0.584	0.424	0.491	0.84	0.621	2.524
	NRAGE-W	107	0.421	0.628	0.671	0.526	0.842	0.666	0.692	0.396	0.504	0.625	0.459	0.529	0.95	0.684	2.89

dataset	mehtods	#clusters	MMR	FRM	SEP	PPV	SN	ACC	Prec	Rec	F-measure	Prec+	Rec+	F-measure	F_MMR	Modularity	Composite Score
	MCL	129	0.496	0.429	0.467	0.448	0.595	0.516	0.637	0.364	0.463	0.749	0.364	0.49	0.986	0.668	2.371
	MCODE	59	0.226	0.335	0.325	0.512	0.338	0.416	0.611	0.219	0.322	0.648	0.228	0.337	0.563	0.479	1.624
	CFinder	62	0.293	0.295	0.334	0.467	0.594	0.527	0.829	0.277	0.415	0.825	0.238	0.369	0.662	0.564	1.864
KroganCo	ClusterOn	144	0.421	0.468	0.418	0.537	0.662	0.596	0.768	0.354	0.485	0.628	0.367	0.463	0.884	0.554	2.388
	ProRank+	265	0.354	0.329	0.195	0.395	0.607	0.49	0.695	0.267	0.386	0.317	0.214	0.256	0.61	0.327	1.754
	CLE	57	0.295	0.361	0.167	0.337	0.309	0.323	0.338	0.339	0.338	0.338	0.246	0.285	0.58	0.292	1.484
	CW	96	0.317	0.327	0.226	0.297	0.329	0.313	0.267	0.428	0.329	0.526	0.318	0.396	0.713	0.338	1.512
	CLP	117	0.364	0.295	0.208	0.339	0.261	0.297	0.485	0.239	0.32	0.498	0.276	0.355	0.719	0.402	1.484
	CI	93	0.267	0.267	0.167	0.418	0.297	0.352	0.567	0.198	0.294	0.367	0.304	0.333	0.6	0.318	1.347
	PC2P	277	0.486	0.664	0.495	0.494	0.668	0.574	0.597	0.618	0.607	0.568	0.437	0.494	0.98	0.589	2.826
	DPCT	165	0.442	0.504	0.398	0.504	0.584	0.543	0.612	0.414	0.494	0.578	0.412	0.481	0.923	0.537	2.381
	NRAGE-W	247	0.428	0.637	0.537	0.528	0.567	0.547	0.558	0.567	0.562	0.596	0.448	0.512	0.94	0.535	2.711

dataset	mehtods	#clusters	MMR	FRM	SEP	PPV	SN	ACC	Prec	Rec	F-measure	Prec+	Rec+	F-measure	F_MMR	Modularity	Composite Score
	MCL	84	0.218	0.267	0.584	0.243	0.815	0.445	0.668	0.208	0.317	0.679	0.208	0.318	0.536	0.295	1.831
	MCODE	51	0.162	0.218	0.423	0.349	0.364	0.356	0.647	0.175	0.275	0.531	0.167	0.254	0.416	0.326	1.434
	CFinder	47	0.227	0.198	0.384	0.261	0.562	0.383	0.728	0.207	0.322	0.548	0.159	0.246	0.473	0.294	1.514
KroganExt	ClusterOn	103	0.267	0.337	0.448	0.561	0.477	0.517	0.857	0.345	0.492	0.715	0.303	0.426	0.693	0.383	2.061
	ProRank+	167	0.195	0.278	0.218	0.368	0.465	0.414	0.728	0.234	0.354	0.354	0.206	0.26	0.455	0.225	1.459
	CLE	69	0.236	0.327	0.228	0.347	0.418	0.381	0.548	0.295	0.384	0.298	0.167	0.214	0.45	0.271	1.556
	CW	93	0.198	0.228	0.264	0.267	0.339	0.301	0.507	0.278	0.359	0.318	0.216	0.257	0.455	0.227	1.35
	CLP	97	0.108	0.239	0.384	0.228	0.384	0.296	0.468	0.262	0.336	0.234	0.223	0.228	0.336	0.265	1.363
	CI	86	0.167	0.348	0.318	0.234	0.309	0.269	0.337	0.282	0.307	0.243	0.276	0.258	0.425	0.74	1.409
	PC2P	274	0.369	0.657	0.502	0.484	0.625	0.55	0.468	0.472	0.47	0.372	0.459	0.411	0.78	0.495	2.548
	DPCT	129	0.312	0.387	0.451	0.446	0.524	0.483	0.584	0.411	0.482	0.394	0.416	0.405	0.717	0.375	2.115
	NRAGE-W	302	0.354	0.538	0.465	0.462	0.573	0.515	0.629	0.394	0.485	0.445	0.383	0.412	0.766	0.384	2.357

Comparative analysis   Human-Corum

dataset	mehtods	#clusters	MMR	FRM	SEP	PPV	SN	ACC	Prec	Rec	F-measure	Prec+	Rec+	F-measure	F_MMR	Modularity	Composite Score
	MCL	373	0.125	0.438	0.194	0.105	0.783	0.287	0.673	0.382	0.487	0.567	0.072	0.128	0.253	0.684	1.531
	MCODE	129	0.067	0.213	0.193	0.137	0.405	0.236	0.708	0.143	0.238	0.669	0.038	0.072	0.139	0.534	0.947
	CFinder	94	0.045	0.257	0.154	0.096	0.496	0.218	0.809	0.325	0.464	0.723	0.046	0.086	0.131	0.634	1.138
STRING	ClusterOn	264	0.043	0.363	0.167	0.087	0.663	0.24	0.628	0.354	0.453	0.583	0.056	0.102	0.145	0.683	1.266
	ProRank+	468	0.098	0.329	0.103	0.092	0.612	0.237	0.673	0.223	0.335	0.451	0.064	0.112	0.21	0.324	1.102
	CLE	159	0.102	0.189	0.068	0.063	0.341	0.147	0.236	0.239	0.237	0.361	0.103	0.16	0.262	0.315	0.743
	CW	113	0.067	0.168	0.073	0.052	0.332	0.131	0.432	0.233	0.303	0.236	0.062	0.098	0.165	0.216	0.742
	CLP	137	0.052	0.203	0.096	0.041	0.523	0.146	0.352	0.359	0.355	0.236	0.063	0.099	0.151	0.193	0.852
	CI	126	0.028	0.216	0.037	0.117	0.439	0.227	0.185	0.392	0.251	0.259	0.071	0.111	0.139	0.203	0.759
	PC2P	483	0.086	0.447	0.183	0.152	0.693	0.325	0.538	0.495	0.516	0.483	0.084	0.143	0.229	0.693	1.557
	DPCT	297	0.038	0.412	0.097	0.084	0.571	0.281	0.497	0.361	0.418	0.492	0.061	0.109	0.147	0.492	1.246
	NRAGE-W	397	0.125	0.504	0.254	0.136	0.673	0.303	0.629	0.525	0.572	0.501	0.106	0.175	0.3	0.664	1.758

Comparative analysis Human-Corum																	
dataset	mehtods	#clusters	MMR	FRM	SEP	PPV	SN	ACC	Prec	Rec	F-measure	Prec+	Rec+	F-measure	F_MMR	Modularity	Composite Score
	MCL	86	0.021	0.124	0.098	0.068	0.835	0.238	0.694	0.085	0.151	0.438	0.009	0.018	0.039	0.267	0.632
	MCODE	57	0.012	0.071	0.083	0.054	0.315	0.13	0.382	0.013	0.025	0.317	0.006	0.012	0.024	0.257	0.321
	CFinder	49	0.016	0.056	0.078	0.039	0.562	0.148	0.526	0.018	0.035	0.392	0.015	0.029	0.045	0.336	0.333
PIPS	ClusterOn	94	0.013	0.146	0.095	0.048	0.468	0.15	0.364	0.039	0.07	0.268	0.009	0.017	0.03	0.386	0.474
	ProRank+	194	0.024	0.064	0.043	0.025	0.483	0.11	0.371	0.086	0.14	0.228	0.014	0.026	0.05	0.118	0.381
	CLE	117	0.019	0.102	0.079	0.036	0.462	0.129	0.426	0.032	0.06	0.134	0.012	0.022	0.041	0.126	0.389
	CW	79	0.008	0.114	0.067	0.038	0.367	0.118	0.461	0.027	0.051	0.167	0.004	0.008	0.016	0.14	0.358
	CLP	84	0.008	0.135	0.082	0.039	0.4	0.125	0.395	0.043	0.078	0.182	0.025	0.044	0.052	0.089	0.428
	CI	91	0.011	0.097	0.067	0.029	0.219	0.08	0.317	0.028	0.051	0.162	0.011	0.021	0.032	0.162	0.306
	PC2P	286	0.046	0.234	0.095	0.074	0.627	0.215	0.638	0.412	0.501	0.454	0.045	0.082	0.128	0.385	1.091
	DPCT	103	0.025	0.218	0.074	0.045	0.512	0.152	0.401	0.059	0.103	0.342	0.028	0.052	0.077	0.448	0.572
	NRAGE-W	301	0.089	0.395	0.097	0.061	0.572	0.187	0.517	0.338	0.409	0.392	0.042	0.076	0.165	0.369	1.177

Supplementary Table 2 The average of enrichment score of predicted complexes with at least one enriched annotation over all clusters are compared among eleven methods across six datasets

Yeast				
	method	BP	MF	CC
Collins	MCL	0.825	0.402	0.526
	MCODE	0.929	0.487	0.638
	CFinder	0.948	0.496	0.623
	ClusterOne	0.953	0.512	0.654
	ProRank+	0.948	0.764	0.721
	CLE	0.817	0.543	0.556
	CW	0.909	0.528	0.608
	CLP	0.899	0.463	0.537
	CI	0.922	0.526	0.519
	PC2P	0.947	0.592	0.685
	DPCT	0.884	0.534	0.612
	NRAGE-W	0.928	0.575	0.657
	method	BP	MF	CC
Gavin	MCL	0.884	0.383	0.693
	MCODE	0.863	0.524	0.697
	CFinder	0.898	0.517	0.701
	ClusterOne	0.905	0.539	0.724
	ProRank+	0.927	0.694	0.766
	CLE	0.925	0.627	0.693
	CW	0.916	0.613	0.719
	CLP	0.936	0.636	0.684
	CI	0.905	0.597	0.707
	PC2P	0.948	0.612	0.782
	DPCT	0.918	0.569	0.704
	NRAGE-W	0.926	0.593	0.729
	method	BP	MF	CC

KroganCo	MCL	0.753	0.406	0.395
	MCODE	0.901	0.539	0.587
	CFinder	0.934	0.594	0.693
	ClusterOn	0.874	0.466	0.718
	ProRank+	0.949	0.691	0.725
	CLE	0.863	0.743	0.984
	CW	0.874	0.756	0.698
	CLP	0.905	0.782	0.706
	CI	0.878	0.846	0.682
	PC2P	0.937	0.946	0.765
	DPCT	0.908	0.714	0.697
	NRAGE-W	0.913	0.896	0.691
	method	BP	MF	CC
KroganExt	MCL	0.827	0.426	0.539
	MCODE	0.865	0.692	0.68
	CFinder	0.902	0.513	0.593
	ClusterOn	0.886	0.654	0.604
	ProRank+	0.846	0.572	0.638
	CLE	0.825	0.638	0.625
	CW	0.847	0.593	0.584
	CLP	0.827	0.741	0.576
	CI	0.839	0.638	0.537
	PC2P	0.853	0.694	0.625
	DPCT	0.819	0.528	0.624
	NRAGE-W	0.852	0.639	0.648
	Human			
	method	BP	MF	CC
STRING	MCL	0.889	0.416	0.713
	MCODE	0.892	0.482	0.734
	CFinder	0.903	0.564	0.856
	ClusterOn	0.893	0.553	0.813

	ProRank+	0.926	0.589	0.844
	CLE	0.904	0.552	0.693
	CW	0.875	0.493	0.705
	CLP	0.862	0.481	0.684
	CI	0.874	0.511	0.796
	PC2P	0.923	0.517	0.834
	DPCT	0.905	0.487	0.841
	NRAGE-W	0.897	0.492	0.865
	Human			
	method	BP	MF	CC
PIPS	MCL	0.837	0.509	0.551
	MCODE	0.869	0.546	0.749
	CFinder	0.957	0.619	0.796
	ClusterOn	0.934	0.627	0.717
	ProRank+	0.962	0.628	0.834
	CLE	0.903	0.592	0.81
	CW	0.897	0.557	0.79
	CLP	0.884	0.573	0.834
	CI	0.904	0.544	0.816
	PC2P	0.936	0.667	0.762
	DPCT	0.895	0.592	0.708
	NRAGE-W	0.919	0.638	0.779
